# Weight of evidence approach using a *TK* gene mutation assay with human TK6 cells for follow-up of positive results in Ames tests: a collaborative study by MMS/JEMS

**DOI:** 10.1186/s41021-021-00179-1

**Published:** 2021-03-06

**Authors:** Manabu Yasui, Takayuki Fukuda, Akiko Ukai, Jiro Maniwa, Tadashi Imamura, Tsuneo Hashizume, Haruna Yamamoto, Kaori Shibuya, Kazunori Narumi, Yohei Fujiishi, Emiko Okada, Saori Fujishima, Mika Yamamoto, Naoko Otani, Maki Nakamura, Ryoichi Nishimura, Maya Ueda, Masayuki Mishima, Kaori Matsuzaki, Akira Takeiri, Kenji Tanaka, Yuki Okada, Munehiro Nakagawa, Shuichi Hamada, Akihiko Kajikawa, Hiroshi Honda, Jun Adachi, Kentaro Misaki, Kumiko Ogawa, Masamitsu Honma

**Affiliations:** 1 Division of Genetics and Mutagenesis, National Institute of Health Sciences, 3-25-26 Tono-machi, Kawasaki-ku, Kawasaki, Kanagawa 210-9501 Japan; 2 Tokyo Laboratory, BoZo Research Center Inc., 1-3-11, Hanegi, Setagaya-ku, Tokyo 156-0042 Japan; 3 AstraZeneca KK, 3-1 Ofuka-cho, Kita-ku, Osaka, 530-0011 Japan; 4 Ina Research Inc., 2148-188 Nishiminowa, Ina-shi, Nagano 399-4501 Japan; 5 Scientific Product Assessment Center, R&D Group, Japan Tobacco Inc., 6-2, Umegaoka, Aoba-ku, Yokohama, Kanagawa 227-8512 Japan; 6 Yakult Central Institute, 5-11 Izumi, Kunitachi-shi, Tokyo 186-8650 Japan; 7 Chemicals Evaluation and Research Institute, Japan, 3-822, Ishii-machi, Hita-shi, Oita 877-0061 Japan; 8 Astellas Pharma Inc., 21, Miyukigaoka, Tsukuba-shi, Ibaraki 305-8585 Japan; 9 Genotoxicology Laboratory, BioSafety Research Center Inc., 582-2 Shioshinden, Iwata-shi, Shizuoka 437-1213 Japan; 10 Chugai Pharmaceutical Co., Ltd, 1-135, Komakado, Gotemba, Shizuoka 412-8513 Japan; 11 Toxicology Research Department, Teijin Institute for Bio-medical Research, Teijin Pharma Limited, 4-3-2, Asahigaoka, Hino, Tokyo 191-8512 Japan; 12 Nonclinical Research Center, LSI Medience Corporation, 14-1, Sunayama, Kamisu-shi, Ibaraki 314-0255 Japan; 13 R&D Safety Science Research, Kao Corporation, Haga–Gun, Tochigi Japan; 14 Laboratory of Proteomics for Drug Discovery, Center for Drug Design Research, National Institutes of Biomedical Innovation, Health and Nutrition, 7-6-8 Saito-Asagi, Ibarak, Osaka 567-0085 Japan; 15 School of Nursing, University of Shizuoka, 52-1 Yada, Suruga-ku, Shizuoka, 422-8526 Japan; 16 Division of Pathology, National Institute of Health Sciences, 3-25-26 Tono-machi, Kawasaki-ku, Kawasaki, Kanagawa 210-9501 Japan

**Keywords:** TK6 assay, Human lymphoblastoid TK6 cells, Ames test, Follow-up, Weight of evidence approach, Toxicoproteomics

## Abstract

**Background:**

Conflicting results between bacterial mutagenicity tests (the Ames test) and mammalian carcinogenicity tests might be due to species differences in metabolism, genome structure, and DNA repair systems. Mutagenicity assays using human cells are thought to be an advantage as follow-up studies for positive results in Ames tests. In this collaborative study, a thymidine kinase gene mutation study (TK6 assay) using human lymphoblastoid TK6 cells, established in OECD TG490, was used to examine 10 chemicals that have conflicting results in mutagenicity studies (a positive Ames test and a negative result in rodent carcinogenicity studies).

**Results:**

Two of 10 test substances were negative in the overall judgment (20% effective as a follow-up test). Three of these eight positive substances were negative after the short-term treatment and positive after the 24 h treatment, despite identical treatment conditions without S9. A toxicoproteomic analysis of TK6 cells treated with 4-nitroanthranilic acid was thus used to aid the interpretation of the test results. This analysis using differentially expressed proteins after the 24 h treatment indicated that in vitro specific oxidative stress is involved in false positive response in the TK6 assay.

**Conclusions:**

The usefulness of the TK6 assay, by current methods that have not been combined with new technologies such as proteomics, was found to be limited as a follow-up test, although it still may help to reduce some false positive results (20%) in Ames tests. Thus, the combination analysis with toxicoproteomics may be useful for interpreting false positive results raised by 24 h specific reactions in the assay, resulting in the more reduction (> 20%) of false positives in Ames test.

**Supplementary Information:**

The online version contains supplementary material available at 10.1186/s41021-021-00179-1.

## Introduction

DNA reactive substances may directly damage DNA even when present at low levels leading to mutations and, therefore, potentially initiating cancer. A positive result in the Ames test has a significant influence on the development of new drugs and chemical substances, and in many cases, mutagenic chemicals are dropped from drug/chemical development at an early stage. In the guidelines from the ICH M7 (ICH: International Council for Harmonization of Technical Requirements for Pharmaceuticals for Human Use), Ames-positive substances are considered as DNA reactive substances [1] and require a great deal of labor for subsequent development and manufacturing. Continued development of components with Ames-positive results requires in vivo tests, such as transgenic rodent mutation assays as follow-up testings. However, problems such as costs and labor burden of in vivo testing can be prohibitive.

Furthermore, such testing does not fit the 3R principles for animal welfare [2, 3]. Positive results in the Ames test correlate well with carcinogenicity in rodents, with the concordance (Ames-negative and carcinogenic-negative) of approximately 80% [4, 5]. This indicates that there are approximately 20% of chemical substances that are positive in Ames tests and negative for carcinogenicity test. In fact, it is considered that some of these substances are unrelated to human carcinogenesis. For example, fexinidazole, a drug for sleeping sickness, is positive for the Ames test but negative for all in vitro (micronucleus test in human lymphocytes) and in vivo genotoxicity tests (ex vivo unscheduled DNA synthesis in rats; bone marrow micronucleus test in mice). Tweats et al. [6] demonstrated that fexinidazole is metabolically activated by a bacterial-specific nitroreductase reaction and is thus positive in the Ames test alone. Thus, if it is possible to prove that the Ames test positive is a bacteria-specific reaction and has a low risk of carcinogenicity in humans, useless a follow-up testing in whole animals can be avoided.

The consideration of the mode of action is the critical establishment of non-animal testing for the safety evaluation of chemicals. The Organization for Economic Co-operation and Development (OECD) have been vigorously developing “adverse outcome pathway (AOP)” and “integrated approaches to testing and assessment (IATA)” combining in silico and in vitro information based on AOP [7]. The AOP and IATA would contribute to a precise toxicological evaluation based on the weight of evidence (WoE) including genotoxicity [7, 8] and the derivation of regulatory conclusions. Since the in vivo testing is prohibited for the safety evaluation of cosmetic ingredients, the use of a WoE approach based on in vitro testing is relatively advanced in the cosmetic industry [9]. An example is the toxicity evaluation of Basic Brown 17, used as a hair coloring agent for cosmetics. Basic Brown 17 is positive in Ames test, but negative in mutation assays using mouse lymphoma (two loci of *TK* and *HPRT*) and in in vitro micronucleus test using mammalian cells. Furthermore, Basic Brown 17 is negative in the comet assay with 3-D reconstituted human skin cells [10, 11]. Thus, based on WoE [12], Basic Brown 17 can be considered to show negligible potential for in vivo genotoxicity, and no additional testing is reported [10]. Furthermore, omics technologies, such as transcriptome and proteome, play a crucial role in implementing a WoE approach. Ates et al. [13] used an in vitro transcriptomics approach to evaluate the genotoxicity predictions for cosmetic ingredients that were negative by in silico analysis but positive by the Ames test. Transcriptomics data were reported to provide needed mechanistic information for toxicity assessments at the gene expression and metabolic pathway levels. In addition, Kirkland et al. [14, 15] also reported that follow-up in vitro tests, such as for gene expression profiling, may aid significantly in interpretation of the relevance for humans of the in vitro genotoxicity results vis-à-vis in vivo genotoxicity or carcinogenicity. Thus, the WoE approaches are also useful for a follow-up strategy for positive Ames tests, shifting steadily toward new evaluation strategies for human risk while reducing dependence on animal experiments.

Based on the current literature, we focused on the utility of the thymidine kinase gene (*TK*) mutation assay using human TK6 cells for the follow-up of positive Ames tests. Conflicting results between Ames tests and mammalian carcinogenicity tests might be caused by species differences in metabolism, genome structure, and DNA repair systems. TK6 cells express human metabolic enzymes and have a chromatin structure. Furthermore, the cells are positive for p53 protein-related functions (competent DNA repair systems) [16]. TK6 cells might be useful for in a WoE approach to scrutinize positive results in the Ames test. However, little is known about the utility of the assay for this purpose. Thus, the present study used the TK6 assay for the follow-up of Ames test results with 10 non-carcinogenic chemicals that were Ames-positive in a collaborative study with 10 laboratories in Japan. Assays were conducted for a short-term (4 h in the presence and absence of rat liver S9) and long-term (24 h in the absence of S9) treatments. Furthermore, we explored the integration of toxicoproteomics analysis with TK6 assays to help interpret test results and increase the utility of WoE.

## Materials and methods

### Participants and test substances

Ten laboratories in Japan, including pharmaceutical and chemical companies, contract laboratories, and public institutes, conducted TK6 assays for 10 test substances in the collaborative study (Table 1). We purchased the test substances from FUJIFILM Wako Pure Chemical Corp. (Osaka, Japan), Tokyo Chemical Industry Co., LTD. (Tokyo, Japan), or Sigma-Aldrich (St. Louis, USA). Most of the test substances were dissolved in dimethyl sulfoxide (DMSO) as a solvent, except that ethanol was used for 1-nitronaphthalene (Table 1).

**Table 1:** Participantes and test substances in the collaborative study

No.	Participating Laboratories	Investigators	Test Substances	CAS No.	Manufacturer, Lot#	Solvent
1	Ina Research Inc.	Tadashi Imamura	4-(Chloroacetyl)acetanilide	140-49-8	FUJIFILM Wako Pure Chemical Corporation, Lot#KPJ1678	DMSO
2	Japan Tobacco Inc.	Tsuneo Hashizume, Haruna Yamamoto, Kaori Shibuya	2-(Chloromethyl) pyridine HCl	6959-47-3	Tokyo Chemical Industry Co., Ltd., Lot#7BOQI-IC	DMSO
3	Yakult Central Institute	Kazunori Narumi, Yohei Fujiishi, Emiko Okada	2,6-Diaminotoluene	823-40-5	Tokyo Chemical Industry Co., Ltd., Lot#IUL6B	DMSO
4	Chemicals Evaluation and Research Institute, Japan	Saori Fujishima	2,5-Diaminotoluene	95-70-5	Tokyo Chemical Industry Co., Ltd., Lot#QFIJE	DMSO
5	Astellas Pharma Inc.	Mika Yamamoto, Naoko Otani	HC Blue No.2	33229-34-4	SIGMA-ALDRICH, Lot#STBF9635V	DMSO
6	BoZo Research Center Inc.	Takayuki Fukuda, Maki Nakamura, Ryoichi Nishimura, Shuichi Hamada	8-Hydroxyquinoline	148-24-3	Tokyo Chemical Industry Co., Ltd., Lot#5PDSI-RB	DMSO
7	BioSafety Research Center Inc.	Maya Ueda	Iodoform	75-47-8	FUJIFILM Wako Pure Chemical Corporation, Lot#PDH1055	DMSO
8	Chugai Pharmaceutical Co., Ltd	Masayuki Mishima, Kaori Matsuzaki, Akira Takeiri, Kenji Tanaka	4-Nitroanthranilic acid	619-17-0	Tokyo Chemical Industry Co., Ltd., Lot#AGM01-AGMQ	DMSO
9	TEIJIN PHARMA LIMITED	Yuki Okada, Takafumi Kimoto	1-Nitronaphthalene	86-57-7	Tokyo Chemical Industry Co., Ltd., Lot#BGF8A-MD	Ethanol
10	LSI Medience Corporation	Munehiro Nakagawa, Akihiko Kajiwara	4-Nitro-o-phenylenediamine	99-56-9	Tokyo Chemical Industry Co., Ltd., Lot#NMEDH	DMSO

### Cell culture

The human lymphoblast cell line TK6 was purchased from the Japanese Collection of Research Bioresources cell bank and the American Type Culture Collection. Cells were cultured at 37 °C with 5% CO_2_ in an RPMI medium containing 10% horse serum (JRH Bioscience), 200 μg/mL sodium pyruvate (Wako Pure Chemical Industries, Ltd. and Thermo Fisher (Gibco)), 100 U/mL penicillin, and 100 μg/mL streptomycin (Nacalai Tesque Inc. and Thermo Fisher (Gibco)). Reagent manufacturers are not necessarily unified, and some research institutions have procured equivalent reagents from other manufacturers.

### The treatment of test substances and dose finding

In principle, this collaborative study conducted the treatment of test substances, the dose finding, and the main test of TK6 assays, according to the OECD Guideline TG490 [17]. We optimized the protocol for TK6 assay prior to this collaborative study. The laboratory of the National Institute of Health Sciences carefully supported the experimental procedures such as chemical treatments and colony counting for TK6 assay performed by each laboratory. Briefly, test substances were dissolved in an appropriate solvent (DMSO or ethanol) and then serially diluted to prepare final concentrations. Details of 10 test substances are shown in Table 2. In the absence (150 mM KCl) or presence of rat liver S9 mix (final concentration 4.5%), cells (2 × 10^7^ cells) were exposed to a test substance and cultured for 4 h. Rat liver S9 was purchased from Oriental Yeast Co., Ltd. and IEDA TRADING Corp. In cases where the short-term treatment (4 h) showed negative results, the 24 h treatment without S9 mix was conducted. Cyclophosphamide (CP, Fujifilm Wako Pure Chemical Industries, Ltd.) was used as a positive control (2.5–3 μg/mL) for metabolic activation, and methyl methanesulfonate (MMS, Tokyo Chemical Industry Co., Ltd., and Sigma Aldrich) was used as a positive control (3–5 μg/mL for 4 h and 1–2.5 μg/mL for 24 h treatments) for non-metabolic activation. The 4 h and 24 h treatments were conducted by shaking and static exposures, respectively. After treatment with test substances, cells were centrifuged (1000 rpm, 5 min), supernatants were removed, and cells were washed with a serum-free medium or Hank’s Balanced Salt Solution. After centrifugation (1000 rpm, 5 min) to remove the supernatant, cells (2 × 10^7^ cells) were dispersed in an RPMI medium containing 10% serum, and cell concentration was measured. Treated cells were cultured at 37 °C with 5% CO_2_ condition and used for TK6 assays. At that time, to calculate the cloning efficiency (CE) (Eq. 1), the cells were cultured in a 96-well microplate at a concentration of about 1.6 cells/well for 2 weeks.

**Table 2:** Testing chemicals for the positive results in bacterial reverse mutation assay and the negative results in carcinogenicity studies^a^

No.	Chemical Name	The Positive Results in Bacterial Reverse Mutation Assay				Carcinogenicity Studies	
		Without S9	With S9	The Highest Specific Activity^b^ and the Strain	Ref.		Ref.
1	4-(Chloroacetyl)-acetanilide	Neg	Pos	600 revertants/mg in TA1538 with S9	[18, 19]	Neg	[20]
2	2-(Chloromethyl) pyridine HCl	Pos	Pos	98.6 revertants/mg in TA100 with S9	[21]	Neg	[22]
3	2,6-Diaminotoluene	Neg	Pos	1703 revertants/mg in TA100 with S9	[23]	Neg	[24]
4	2,5-Diaminotoluene	Neg	Pos	8060 revertants/mg in TA98 and 3840 revertants/mg in TA100 with S9	[25]	Neg	[26]
5	HC Blue No.2	Pos	Pos	153.6 revertants/mg in TA98 with S9	[27, 28]	Neg	[29]
6	8-Hydroxyquinoline	Neg	Pos	21,400 revertants/mg in TA97 and 16,000 revertants/mg in TA100 with S9	[28]	Neg	[30]
7	Iodoform	Pos	Pos	465 revertants/mg in TA98 and 450 revertants/mg in TA100 with S9	[31]	Neg	[32]
8	4-Nitroanthranilic acid	Pos	Pos	3600 revertants/mg in TA1535 and 2830 revertants/mg in TA100 with S9	[33]	Neg	[34]
9	1-Nitronaphthalene	Pos	Pos	14,700 revertants/mg in TA100 without S9 and 12,500 revertants/mg in TA100 with S9	[35]	Neg	[36]
10	4-Nitro-o-phenylenediamine	Pos	Pos	41,400 revertants/mg in TA100 without S9	[37–40]	Neg	[41]

^a^*Pos* Positive, *Neg* Negative

^b^Specific activity value indicating that strong mutagenicity is observed in the Ames test is approximately 1000 (revertants / mg) or more, and to be calculated as below

Specific activity (revertants / mg) = {(Number of colonies per plate at the dose value) - (Number of colonies per plate in negative control test)}/The dose value (μg) X 1000

CE, the cell colony formation rate, was calculated using Eq. 1 according to the Poisson distribution equation. EW was the number of wells without colonies, and TW was the total number of wells. N was the average number of cells per well (*N* = 1.6).

1 $$ \mathrm{CE}=-\ln\ \left(\mathrm{EW}/\mathrm{TW}\right)/\mathrm{N} $$

Additionally, CE was adjusted by the following calculation (Eq. 2) due to cell loss when exposure caused cytotoxicity. “The number of cells at the end of the treatment” was the number of cells obtained after centrifugation at the end of the treatment in the above-described exposure treatment of the test substance. The “cell number at the start of treatment” was 2 × 10^7^.

2 $$ \mathrm{Adjusted}\ \mathrm{CE}=\mathrm{CE}\times \mathrm{number}\ \mathrm{of}\ \mathrm{cells}\ \mathrm{at}\ \mathrm{end}\ \mathrm{of}\ \mathrm{treatment}/\mathrm{number}\ \mathrm{of}\ \mathrm{cells}\ \mathrm{at}\ \mathrm{the}\ \mathrm{start}\ \mathrm{of}\ \mathrm{treatment} $$

CE0 seeding, in which cells were seeded immediately after treatment, and CE3 seeding, in which cells were seeded 3 days after treatment, to examine cell viability in the TK6 assay described below. The relative cell viability RS0 (%) just after the treatment with the test substance was calculated from CE0, following Eq. 3. The survival rate of the negative control group was defined as 100%.

3 $$ \mathrm{RS}0\ \left(\%\right)=\mathrm{adjusted}\ \mathrm{CE}0\ \mathrm{of}\ \mathrm{treatment}\ \mathrm{culture}/\mathrm{adjusted}\ \mathrm{CE}0\ \mathrm{of}\ \mathrm{solvent}\ \mathrm{control}\times 100 $$

### The main test of the TK6 assay

The TK6 assay is conducted between 20 and 10% RS0 as a maximum dose if cytotoxicity is observed. If no precipitate or limiting cytotoxicity was observed, the maximum dose was the lowest concentration among 10 mM, 2 mg/mL, or 2 μL/mL of the test substance. When the main TK6 assay was difficult to conduct under the condition of RS0 values from 20 to 10%, the test was conducted between 20 and 10% relative total growth (RTG), described later, as a maximum dose.

Besides RS0, relative suspension growth (RSG) and RTG as other cytotoxicity indices (Eq. 4) were used. When cells were cultured for 3 days after treatment with the test substance, suspension cell growth ratio 1 (SG1) was the growth ratio from day 0 to day 1 (cell concentration on day 1/cell concentration on day 0). The suspension cell growth ratio 2 (SG2) was the growth ratio from day 1 to day 2 (cell concentration on day 2/cell concentration on day 1). The RSG value was calculated by dividing the total SG value of the untreated group for 3 days by the total SG value of the treated group for the same 3 days (Eq. 4).

On the third day of culture, CE3 plates for determining plating efficiency and mutant frequency (MF) plates for mutation detection were prepared. CE3 plates were seeded in 96-well microplates at a concentration of about 1.6 cells/well. The MF plates were seeded in 96-well microplates at 40,000 cells/well in the presence of 3 μg/mL of trifluorothymidine (Sigma Aldrich).

4 $$ \mathrm{RSG}=\left[\mathrm{SG}1\ \left(\mathrm{treated}\right)\times \mathrm{SG}2\ \left(\mathrm{treated}\right)\times \mathrm{SG}3\ \left(\mathrm{treated}\right)\right]/\left[\mathrm{SG}1\ \left(\mathrm{control}\right)\times \mathrm{SG}2\ \left(\mathrm{control}\right)\times \mathrm{SG}3\ \left(\mathrm{control}\right)\right] $$

5 $$ \mathrm{RTG}\ \left(\%\right)=\mathrm{RSG}\times \mathrm{RS}3 $$

6 $$ \mathrm{RS}3\ \left(\%\right)=\mathrm{treated}\ \mathrm{CE}3/\mathrm{control}\ \mathrm{CE}3\times 100 $$

Mutant colonies in MF plates were calculated using Eq. 7 using the Poisson distribution. EW was the number of wells without colonies, and TW was the total number of wells. N was the average number of cells per well (*N* = 40,000 in this study). The data were statistically analyzed by Omori’s method, a modified Dunnett’s procedure for identifying clear negatives, a Simpson–Margolin procedure for detecting downturn data, and a trend test to evaluate the dose dependency [42, 43]. The acceptability criterion for the test was that the MF value of the positive control group of each laboratory was increased with statistical significance compared with that of the concurrent negative control group.

7 $$ \mathrm{MF}=\left[-\ln\ \left(\mathrm{EW}/\mathrm{TW}\right)/\mathrm{N}\right]/\mathrm{treated}\ \mathrm{CE}3 $$

### Sample preparation for proteomic analysis

We conducted a proteomic analysis using TK6 cells treated by 4-nitroanthranilic acid that was negative in the short-term treatment and positive in the 24 h treatment. HC Blue No. 2 gave also same test results. 4-Nitroanthranilic acid, but not HC Blue No. 2, increased clearly the mutant efficiency in dose-dependent manner even at the low-dose range in the 24 h treatment (Additional file 1 (Table S1 (No.8)), we selected the chemical for the proteomics. Cell treatment with 4-nitroanthranilic acid was the same as the short-time treatment (4 h, −S9mix) and long-term treatment (24 h, −S9mix) in the TK6 assay. Treatment concentrations were 0, 400, and 800 μg/mL, relatively low toxicity doses, to clearly measure biological responses to the chemical. After treatment, the supernatant was removed by centrifugation (1000 rpm, 5 min). The cell pellet (6 × 10^6^ cells) was washed with a 10 mL of ice-cold phosphate-buffered saline (PBS) and centrifuged (repeated twice on ice throughout). The cells were resuspended in 3 mL of PBS containing the protease inhibitor cocktail (Roche), complete EDTA-free (Roche), and 2 × 10^6^ cells each were dispensed into three pre-cooled 2.0 mL tubes. After centrifugation, supernatants were removed again, and tubes containing cell pellets were immersed in liquid nitrogen to freeze the cell pellet and stored at − 80 °C.

Each frozen cell pellet was mixed with PTS (phase transfer surfactant) buffer and boiled at 95 °C for 5 min [44]. Lysates were further sonicated three times (15 min per cycle) with a Bioruptor sonicator (Cosmo Bio, Tokyo, Japan). Samples were then reduced with 10 mM TCEP (tris(2-carboxyethyl) phosphine), alkylated with 20 mM iodoacetamide, and sequentially, digested with trypsin (protein weight: 1/50) and Lys-C (protein weight: 1/50) for 16 h at 37 °C. Peptides were elucidated by centrifugation for 10 min at 20,000 *g* and desalted on a C18-SCX StageTips [45].

### LC-MS/MS analysis

LC-MS/MS was conducted by coupling an UltiMate 3000 Nano LC system (Thermo Scientific, Bremen, Germany) and an HTC-PAL autosampler (CTC Analytics, Zwingen, Switzerland) to a Q Exactive hybrid quadrupole-Orbitrap mass spectrometer (Thermo Scientific). Peptides were loaded on an analytical column (75 μm × 30 cm, packed in-house with ReproSil-Pur C18-AQ, 1.9 μm resin, Dr. Maisch, Ammerbuch, Germany) and separated at a flow rate of 280 nL/min using a 45 min gradient from 5 to 35% of solvent B (solvent A, 0.1% FA and 2% acetonitrile; solvent B, 0.1% FA and 90% acetonitrile). The Q Exactive instrument was operated in the data-dependent mode. Survey full-scan MS spectra (m/z 350–1800) were acquired in the Orbitrap with 70,000 resolution after the accumulation of ions to a 3 × 10^6^ target value. Dynamic exclusion was set to 10 s. The 12 most intense multiplied charged ions (z ≥ 2) were sequentially accumulated to a 1 × 10^5^ target value and fragmented in the collision cell by higher-energy collisional dissociation (HCD) with a maximum injection time of 120 ms and 35,000 resolution. Typical mass spectrometric conditions were: spray voltage, 2 kV; heated capillary temperature, 250 °C; and normalized HCD collision energy, 25%. The MS/MS ion selection threshold was set to 2.5 × 10^4^ counts. A 2.0 Da isolation width was chosen.

Raw MS data were processed by MaxQuant (version 1.6.3.3) supported by the Andromeda search engine for peak detection and quantification. MS/MS spectra were searched against the UniProt human database with the following search parameters: full tryptic specificity, up to two missed cleavage sites, carbamidomethylation of cysteine residues set as a fixed modification, and N-terminal protein acetylation and methionine oxidation as variable modifications. Search results were filtered to a maximum FDR (false discovery rate) of 0.01 at protein, and PSM levels.

### Extraction of differentially expressed proteins

All NaN values of log_2_ LFQ intensities were converted to − 7, which is approximately equivalent to the minimum value of the LFQ intensities observed in the present study. Then, fold change values were calculated by dividing LFQ intensities (antilogarithms of the log_2_ LFQ intensities) into sample treated groups by intensities in non-treated (control) groups, respectively (4 and 24 h treated groups). Then, log_2_ fold changes were calculated. Finally, differentially expressed proteins (DEPs) that were notably expressed in each sample treated group were identified based on log_2_ fold change of ≥2. Next, to analyze specific effects in repeated treatments at the highest dose, DEPs in 24 h 800 μg/mL group was subjected to further screening based on log_2_ fold change between 24-h 800 μg/mL group and other groups ≥1. Thereby, the specific DEPs in 24 h 800 μg/mL group were thus identified. Log_2_ fold changes in LFQ intensities were used to generate a heatmap using the R package (heatmap3).

### Enrichment analysis

Enrichment analysis was used to interpret the biological processes and molecular functions of DEPs. DAVID bioinformatics v6.8 (https://david.ncifcrf.gov/) was used for enrichment analyses to annotate DEPs to their correlated GO (Gene Ontology) terms and pathways. Specifically, Gene Ontology (GOTERM_BP_DIRECT, GOTERM_CC_DIRECT, GOTERM_MF_DIRECT), and Protein_Domains (INTERPRO, PIR_SUPERFMILY, SMART) were analyzed. Moreover, since DNA damage and oxidative stress were considered as important mechanisms to interpret genotoxicity test results, enrichment scores (the Expression Analysis Systematic Explorer (EASE) *p*-value) of some related terms, such as GO:0006974 (cellular response to DNA damage stimulus) and GO:0006979 (response to oxidative stress) were respectively evaluated even if they were not statistically significant. Statistical significance was expressed as −log (the EASE *p*-value) and was illustrated as bar charts for comparison.

## Results

### Negative and positive control data

**Fig. 1:**
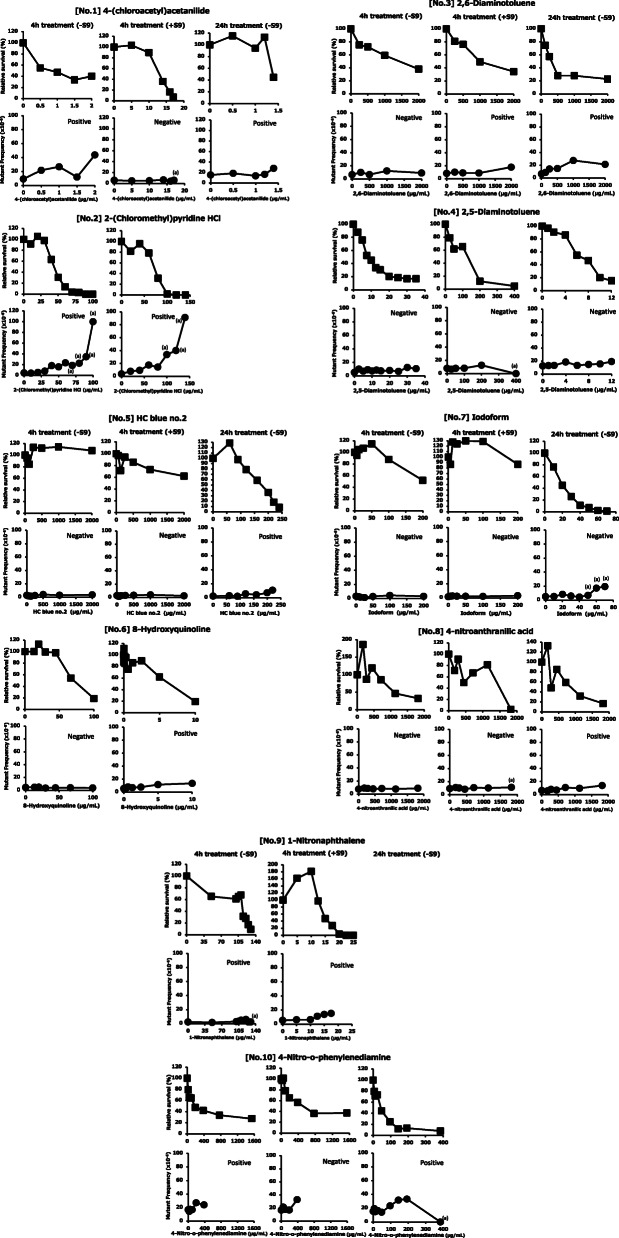
Relative survival and mutant frequency in TK6 assay for the 10 chemicals conducted by each laboratory. According to the OECD guidelines (TG490), dose finding tests were conducted before the main TK6 assays. The short- and long-term treatments were conducted for 4 h (+/−S9mix) and 24 h (−S9mix), respectively. The main tests were conducted so that the highest concentration was set at a concentration where the RS was from 10 to 20%. Experiments and statistical analysis were conducted as described in Materials and Methods. (a) indicates the data of MF were excluded from the statistical judgment because RS was below 10%

The TK6 assay conducted by each laboratory were presented in Fig. 1 (No.1–10) and Table 3. The mutant frequencies of negative control were 2.5 to 16.9 × 10^− 6^ (mean 6.36 × 10^− 6^) for the short treatment without S9 mix, 1.7 to 16.8 × 10^− 6^ (mean 6.78 × 10^− 6^) for the short treatment with S9 mix, and 2.3 to 15.5 × 10^− 6^ (mean 9.09 × 10^− 6^) for the 24 h treatment without S9 mix. According to the laboratory of the National Institute of Health Sciences, the historical spontaneous mutant frequency is 4 to 10 × 10^− 6^, these mean values almost accepted the criteria by OECD TG490 [17]. In addition, the mutant frequencies of the concurrent positive controls were 9.4 to 71.7 × 10^− 6^ (mean 30.1 × 10^− 6^) for MMS during the short treatment without S9 mix, 10.4 to 57.8 × 10^− 6^ (mean 28.6 × 10^− 6^) for CP during the short treatment with S9 mix, and 18.2 to 120 × 10^− 6^ (mean 61.6 × 10^− 6^) for MMS during the 24 h treatment without S9 mix. Thus, the concurrent positive controls produced a statistically significant increase compared with the concurrent negative control. For more details, see Additional file 1 (Table S1 (No.1–10)) attached in this paper. The summary of statistics analysis by Omori’s method in each test substance was provided in Additional file 2 (Table S2).

**Table 3:** Summary of results of *TK* gene mutation assay for the 10 chemicals

No.	Chemical Name	CAS No.	*TK mutation assay*^a^			Bacterial reverse mutation assay^b^		MLA (TK gene locus)		
			Without S9	With S9	Long term	Without S9	With S9	Without S9	With S9	Ref.
1	4-(Chloroacetyl)-acetanilide	140-49-8	Pos	Neg	Pos	Neg	Pos	No data		
2	2-(Chloromethyl) pyridine HCl	6959-47-3	Pos	Pos	NP	Pos	Pos	Pos	Pos	[47]
3	2,6-Diaminotoluene	823-40-5	Neg	Pos	Pos	Neg	Pos	Pos	No data	[48]
4	2,5-Diaminotoluene	95-70-5	Neg	Neg	Neg	Neg	Pos	Inconclusive^c^		[46, 49]
5	HC Blue No.2	33229-34-4	Neg	Neg	Pos	Pos	Pos	Pos	Pos	[27]
6	8-Hydroxyquinoline	148-24-3	Neg	Pos	NP	Neg	Pos	Pos	No data	[50]
7	Iodoform	75-47-8	Neg	Neg	Neg	Pos	Pos	No data		
8	4-Nitroanthranilic acid	619-17-0	Neg	Neg	Pos	Pos	Pos	Equivocal^d^	Pos	[51]
9	1-Nitronaphthalene	86-57-7	Pos	Pos	NP	Pos	Pos	No data		
10	4-Nitro-o-phenylenediamine	99-56-9	Pos	Neg	Pos	Pos	Pos	Pos	Pos	[52, 53]

^a^Performed in this study. *NP* Not applicated

^b^Taken from Table 2

^c^Examined as toluene-2,5-diamine sulfate. The required toxicity (10–20% survival compared to the concurrent negative controls) was not reached in the experiments with S9mix [46]

^d^Significant difference only at the highest dose of 1200 μg/mL [51]

### 4-(Chloroacetyl) acetanilide

4-(Chloroacetyl) acetanilide was prepared by dissolving in DMSO. Dose levels of 4-(chloroacetyl) acetanilide were set at 0.5, 1, 1.5 and 2 μg/mL in the short-term treatment (−S9), 5, 10, 14, 16, and 17 μg/mL with the short-term treatment (+S9), and 0.5, 1.0, 1.2, and 1.4 μg/mL with the long-term treatment based on the results of the dose range-finding tests. A positive response was statistically significant in the Dunnett type test in the short-term (−S9) and long-term treatments. However, no statistically significant linear trends were observed (Additional file 2 (Table S2)). Both MF values clearly exceeded those of the solvent control group. Therefore, based on expert judgment, 4-(chloroacetyl) acetanilide was judged to be positive.

### 2-(Chloromethyl) pyridine HCl

About 80% cytotoxicity (relative colony efficiency) was observed at 60 μg/mL in the absence or at 100 μg/mL in the presence of S9. Regardless of metabolic activation, a clear concentration-dependent increase of MF was observed under the condition used in this study. Long-term treatment was not applied. The test chemical, 2-(chloromethyl) pyridine HCl, was judged as positive.

### 2,6-Diaminotoluene

More than 80% cytotoxicity was observed at 2000 μg/mL in the long-term treatment. Statistically significant increases of MF were observed after the short-term treatment with metabolic activation and after the long-term treatment. Thus, 2,6-diaminotoluene was judged to be positive.

### 2,5-Diaminotoluene

About 80% of cytotoxicity was observed at 20.0 μg/mL in the absence or at 200 μg/mL in the presence of S9 for the short-term treatment and 10.0 μg/mL for the long-term treatment. Statistically significant increases of mutation frequency were not observed after the short-term treatment in the absence or presence of metabolic activation and after the long-term treatment. Thus, 2,5-diaminotoluene was judged to be negative.

### HC Blue No. 2

Based on the results of the dose finding study, concentrations were determined. In the 4 h treatment with and without S9, 62.5–2000 μg/mL were selected. In the 24 h treatment without S9, 60–240 μg/mL were selected. No precipitation was observed at any concentration in all groups. About 80% cytotoxicity was observed at 220 μg/mL in the 24 h treatment without S9 and about 40% cytotoxicity was observed at 2000 μg/mL in 4 h treatment with S9. No cytotoxicity was observed in 4 h treatment without S9. There was no significant difference in MF after 4 h treatment with and without S9. After the 24 h treatment without S9, MF increased in a concentration-dependent manner as compared with that of the solvent control group and showed a statistically significant difference. HC Blue No. 2 was judged to have gene mutagenicity.

### 8-Hydroxyquinoline

After the short-term treatment (+S9), 8-Hydroxyquinoline showed cytotoxicity at the dose level of 10.0 μg/mL, at which the RS was 19.3%. Mutagenic responses to 8-hydroxyquinoline were significant in both Dunnett type test and linear trend tests with S9. After the short-term treatment (−S9), 8-hydroxyquinoline showed cytotoxicity at a dose level of 100 μg/mL, at which the RS was 18.9%. Mutagenic responses to 8-hydroxyquinoline were not significant in the Dunnett type test in without S9. Therefore, based on the above results, it was concluded that 8-hydroxyquinoline had mutagenic potential in TK6 cells under the conditions of this study. Long-term treatment was not applied.

### Iodoform

After the 24 h treatment without S9, Iodoform showed cytotoxicity at the dose level of 40.0 μg/mL, at which the RS was 10.9%. Conversely, after 4 h treatment with/without S9, because of the strong toxicity during the expression period, mutation frequency at the concentration with RS of 10–20% could not be evaluated. Therefore, an additional study was conducted with treatment concentration at which RTG was 10–20% set as maximum concentration. Iodoform showed cytotoxicity at a dose level of 100 (−S9) and 200 (+S9) μg/mL, at which the RTGs were 19.1 and 20.1%, respectively. A dose-dependent increase in MF was observed after the long-term treatment at the high-dose levels (50–70 μg/mL), which showed RS < 10%. These dose levels were excluded from the statistical judgment because of high cytotoxicity. MF values from 10 to 40 μg/mL were significant in the Dunnett modified test (Additional file 2 (Table S2)) but were not increased dose-dependently within the dose range. Hence, we suggest that the significant result obtained from the Dunnett modified test was not biologically relevant in this study. Therefore, the mutagenic potential of iodoform was negative as expert judgment.

### 4-Nitroanthranilic acid

More than 50% cytotoxicity was observed at 1138 and 1821 μg/mL without metabolic activation, and approximately 100% cytotoxicity was observed at 1821 μg/mL with metabolic activation for the short-term treatment. More than 80% cytotoxicity was observed at 1821 μg/mL for the long-term treatment. There was no increase in mutation frequency after the short-term treatment with or without metabolic activation. A statistically significant increase in mutation frequency after the long-term treatment was observed.

### 1-Nitronaphthalene

Doses showing 80% cytotoxicity in the absence of S9 were between 120 and 125 μg/mL. Doses showing 80% cytotoxicity with S9 were between 17.5 and 20 μg/mL. Regardless of metabolic activation and exposure time, a significant increase in MF was observed under the condition used in this study. Long-term treatment was not applied. Hence, this test chemical, 1-nitronaphthalene, was judged as positive.

### 4-Nitro-*o*-phenylenediamine

More than 80% cytotoxicity was observed at 144 μg/mL and above for the long-term treatment. MF was increased significantly in the statistical analysis compared with the negative control value for short-term (−S9) and long-term assays. The judgment for this chemical was positive. Cells exposed to 770 and 1540 μg/mL (+/−S9) were abandoned because of high cytotoxicity.

### Proteomics analysis

**Fig. 2:**
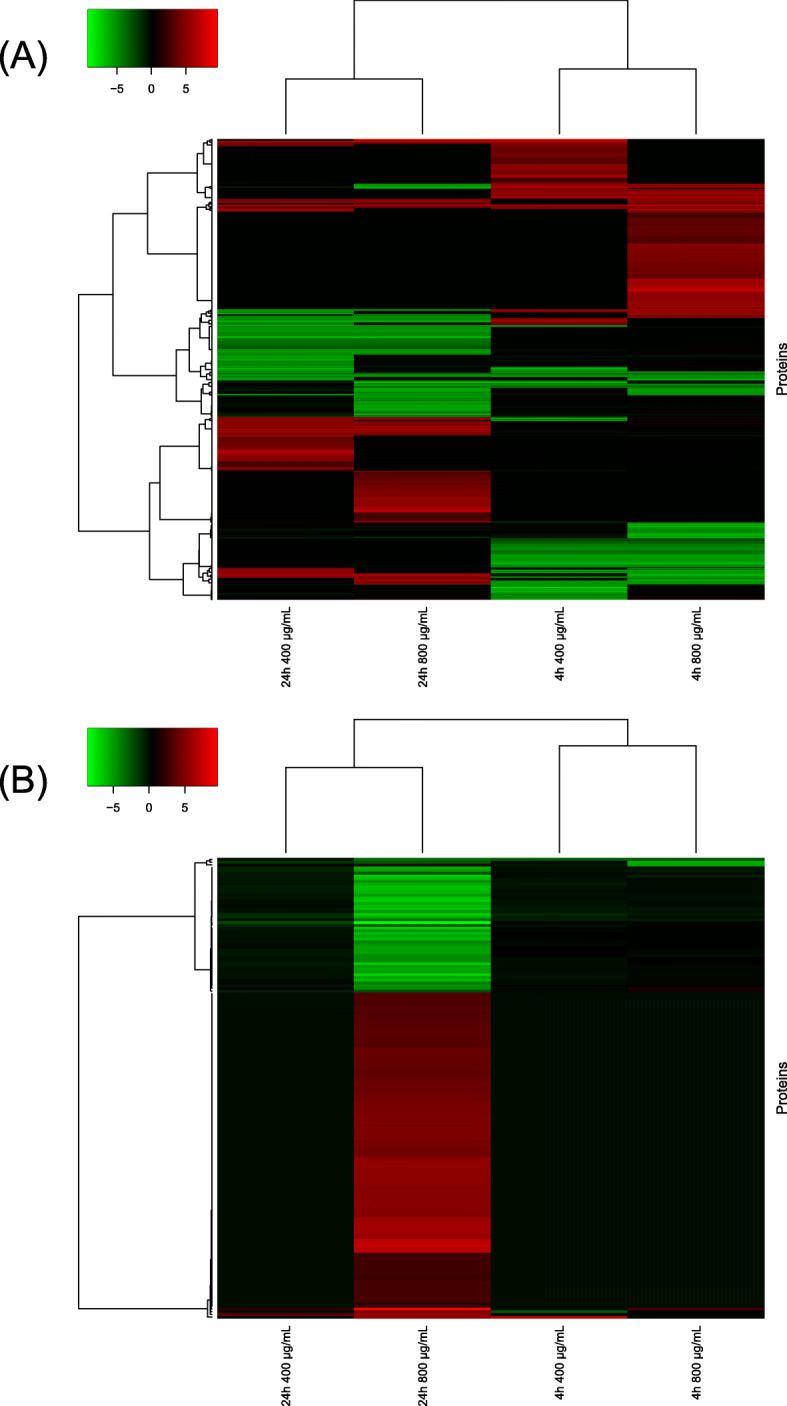
Differentially expressed proteins (DEP) identified in TK6 cells exposed to 4-nitroanthranilic acid. **a** A total of 1078 DEPs that differentially expressed compared to control groups were identified based on a log_2_ fold change of ≥2. Effects of treatment time are greater than that of dose. **b** A total of 168 specific DEPs that differentially expressed compared to other groups were further extracted from the 420 DEPs in 24 h 800 μg/mL group based on a log_2_ fold change of ≥1

A total of 1078 DEPs (4 h 400 μg/mL group: 359 proteins, 4 h 800 μg/mL group: 506 proteins, 24 h 400 μg/mL group: 358 proteins, and 24 h 800 μg/mL group: 420 proteins) were identified based on log_2_ fold change of ≥2 (Fig. 2a). Furthermore, 168 specific DEPs in the 24 h 800 μg/mL group were extracted from 420 DEPs in 24 h 800 μg/mL group based on a log_2_ fold change between 24 h 800 μg/mL group and other groups ≥1 (Fig. 2b).

In the analysis of specific DEPs in 24 h 800 μg/mL group, the enrichment analysis of upregulated proteins revealed that the expression level of proteins that associated with transcription, cellular stress and cell division was remarkably upregulated (e.g., GO:0034244 ~ negative regulation of transcription elongation from RNA polymerase II promoter, GO:0008631 ~ intrinsic apoptotic signaling pathway in response to oxidative stress, GO:0051301 ~ cell division, and GO:2000145 ~ regulation of cell motility) (Table 4A). Furthermore, the analysis of downregulated proteins revealed proteins related to rRNA processing and cell division (e.g., GO:0000462 ~ maturation of SSU-rRNA from tricistronic rRNA transcript (SSU-rRNA, 5.8S rRNA, and LSU-rRNA) and GO:0051301 ~ cell division).

**Table 4:** Enrichment analysis of 168 specific DEPs in the tretment of 4-nitroanthranilic acid (24-h 800 μg/mL group)

Category	Term	Count	*P*Value
A) Up-regulated proteins			
INTERPRO	IPR016239:Ribosomal protein S6 kinase II	4	5.74E-06
GOTERM_MF_DIRECT	GO:0005515 ~ protein binding	88	1.74E-05
PIR_SUPERFAMILY	PIRSF000606:ribosomal protein S6 kinase II	4	3.22E-05
GOTERM_MF_DIRECT	GO:0016301 ~ kinase activity	10	6.80E-05
GOTERM_CC_DIRECT	GO:0005739 ~ mitochondrion	22	2.16E-04
SMART	SM00133:S_TK_X	5	3.29E-04
GOTERM_CC_DIRECT	GO:0005829 ~ cytosol	39	4.31E-04
INTERPRO	IPR000961:AGC-kinase, C-terminal	5	5.33E-04
GOTERM_CC_DIRECT	GO:0005654 ~ nucleoplasm	34	6.58E-04
INTERPRO	IPR017892:Protein kinase, C-terminal	4	0.0010359705
GOTERM_CC_DIRECT	GO:0005759 ~ mitochondrial matrix	9	0.0017034795
GOTERM_BP_DIRECT	GO:0034244 ~ negative regulation of transcription elongation from RNA polymerase II promoter	3	0.0027144316
GOTERM_CC_DIRECT	GO:0005737 ~ cytoplasm	51	0.0027796207
GOTERM_BP_DIRECT	GO:0008631 ~ intrinsic apoptotic signaling pathway in response to oxidative stress	3	0.0050854061
GOTERM_CC_DIRECT	GO:0005840 ~ ribosome	6	0.0053822808
GOTERM_MF_DIRECT	GO:0044822 ~ poly(A) RNA binding	17	0.0078806589
GOTERM_CC_DIRECT	GO:0005743 ~ mitochondrial inner membrane	9	0.0101302596
GOTERM_CC_DIRECT	GO:0005793 ~ endoplasmic reticulum-Golgi intermediate compartment	4	0.0109324145
GOTERM_BP_DIRECT	GO:0001701 ~ in utero embryonic development	6	0.011424021
GOTERM_CC_DIRECT	GO:0016020 ~ membrane	25	0.0115217146
GOTERM_MF_DIRECT	GO:0005524 ~ ATP binding	20	0.0117814813
GOTERM_BP_DIRECT	GO:0010628 ~ positive regulation of gene expression	7	0.0117937932
GOTERM_BP_DIRECT	GO:0051301 ~ cell division	8	0.0134335953
GOTERM_BP_DIRECT	GO:0043555 ~ regulation of translation in response to stress	2	0.0143601269
INTERPRO	IPR027417:P-loop containing nucleoside triphosphate hydrolase	13	0.0146424367
GOTERM_MF_DIRECT	GO:0004672 ~ protein kinase activity	8	0.0161283188
GOTERM_MF_DIRECT	GO:0000287 ~ magnesium ion binding	6	0.0168341212
GOTERM_BP_DIRECT	GO:2000145 ~ regulation of cell motility	3	0.0172254639
INTERPRO	IPR018199:Ribosomal protein S4e, N-terminal, conserved site	2	0.0199116243
INTERPRO	IPR013845:Ribosomal protein S4e, central region	2	0.0199116243
INTERPRO	IPR013843:Ribosomal protein S4e, N-terminal	2	0.0199116243
INTERPRO	IPR000876:Ribosomal protein S4e	2	0.0199116243
INTERPRO	IPR000633:Vinculin, conserved site	2	0.0199116243
GOTERM_BP_DIRECT	GO:0006368 ~ transcription elongation from RNA polymerase II promoter	4	0.0242278191
INTERPRO	IPR001033:Alpha-catenin	2	0.0264610401
INTERPRO	IPR023321:PINIT domain	2	0.0264610401
GOTERM_CC_DIRECT	GO:0032021 ~ NELF complex	2	0.0267274499
GOTERM_MF_DIRECT	GO:0048487 ~ beta-tubulin binding	3	0.0282329504
GOTERM_BP_DIRECT	GO:0019673 ~ GDP-mannose metabolic process	2	0.0285157205
SMART	SM00220:S_TKc	7	0.0318734045
GOTERM_BP_DIRECT	GO:0030521 ~ androgen receptor signaling pathway	3	0.0351748989
GOTERM_MF_DIRECT	GO:0061665 ~ SUMO ligase activity	2	0.0359086364
PIR_SUPERFAMILY	PIRSF002116:ribosomal protein S4a/S4e	2	0.0367953139
INTERPRO	IPR002942:RNA-binding S4 domain	2	0.03942991
GOTERM_CC_DIRECT	GO:0022627 ~ cytosolic small ribosomal subunit	3	0.0416602582
GOTERM_BP_DIRECT	GO:0043620 ~ regulation of DNA-templated transcription in response to stress	2	0.0424696701
GOTERM_BP_DIRECT	GO:0046939 ~ nucleotide phosphorylation	2	0.0424696701
INTERPRO	IPR017441:Protein kinase, ATP binding site	7	0.0427994892
GOTERM_BP_DIRECT	GO:0007507 ~ heart development	5	0.0434212707
GOTERM_MF_DIRECT	GO:0016887 ~ ATPase activity	5	0.0449232427
INTERPRO	IPR000719:Protein kinase, catalytic domain	8	0.0450805987
INTERPRO	IPR006077:Vinculin/alpha-catenin	2	0.0458499332
INTERPRO	IPR004181:Zinc finger, MIZ-type	2	0.0458499332
GOTERM_MF_DIRECT	GO:0003723 ~ RNA binding	9	0.0467405365
B) Down-regulated proteins			
GOTERM_CC_DIRECT	GO:0005730 ~ nucleolus	13	5.68E-07
GOTERM_MF_DIRECT	GO:0044822 ~ poly(A) RNA binding	15	7.76E-07
GOTERM_BP_DIRECT	GO:0006364 ~ rRNA processing	7	1.24E-05
GOTERM_CC_DIRECT	GO:0005634 ~ nucleus	27	4.81E-05
GOTERM_CC_DIRECT	GO:0032040 ~ small-subunit processome	4	8.14E-05
GOTERM_CC_DIRECT	GO:0005737 ~ cytoplasm	25	2.75E-04
GOTERM_CC_DIRECT	GO:0005654 ~ nucleoplasm	17	4.87E-04
GOTERM_BP_DIRECT	GO:0000462 ~ maturation of SSU-rRNA from tricistronic rRNA transcript (SSU-rRNA, 5.8S rRNA, LSU-rRNA)	3	0.0027542237
GOTERM_MF_DIRECT	GO:0005515 ~ protein binding	34	0.0028754728
INTERPRO	IPR006709:Small-subunit processome, Utp14	2	0.0049511535
GOTERM_CC_DIRECT	GO:0005815 ~ microtubule organizing center	4	0.0059735276
GOTERM_BP_DIRECT	GO:0051301 ~ cell division	5	0.0102371094
GOTERM_CC_DIRECT	GO:0034388 ~ Pwp2p-containing subcomplex of 90S preribosome	2	0.0167816086
GOTERM_BP_DIRECT	GO:0002726 ~ positive regulation of T cell cytokine production	2	0.0241562593
GOTERM_CC_DIRECT	GO:0070062 ~ extracellular exosome	13	0.0308651552
GOTERM_BP_DIRECT	GO:0030490 ~ maturation of SSU-rRNA	2	0.0336585264
INTERPRO	IPR017986:WD40-repeat-containing domain	4	0.0400340949
GOTERM_BP_DIRECT	GO:0022904 ~ respiratory electron transport chain	2	0.0477428897
INTERPRO	IPR015943:WD40/YVTN repeat-like-containing domain	4	0.0486054435

**Fig. 3:**
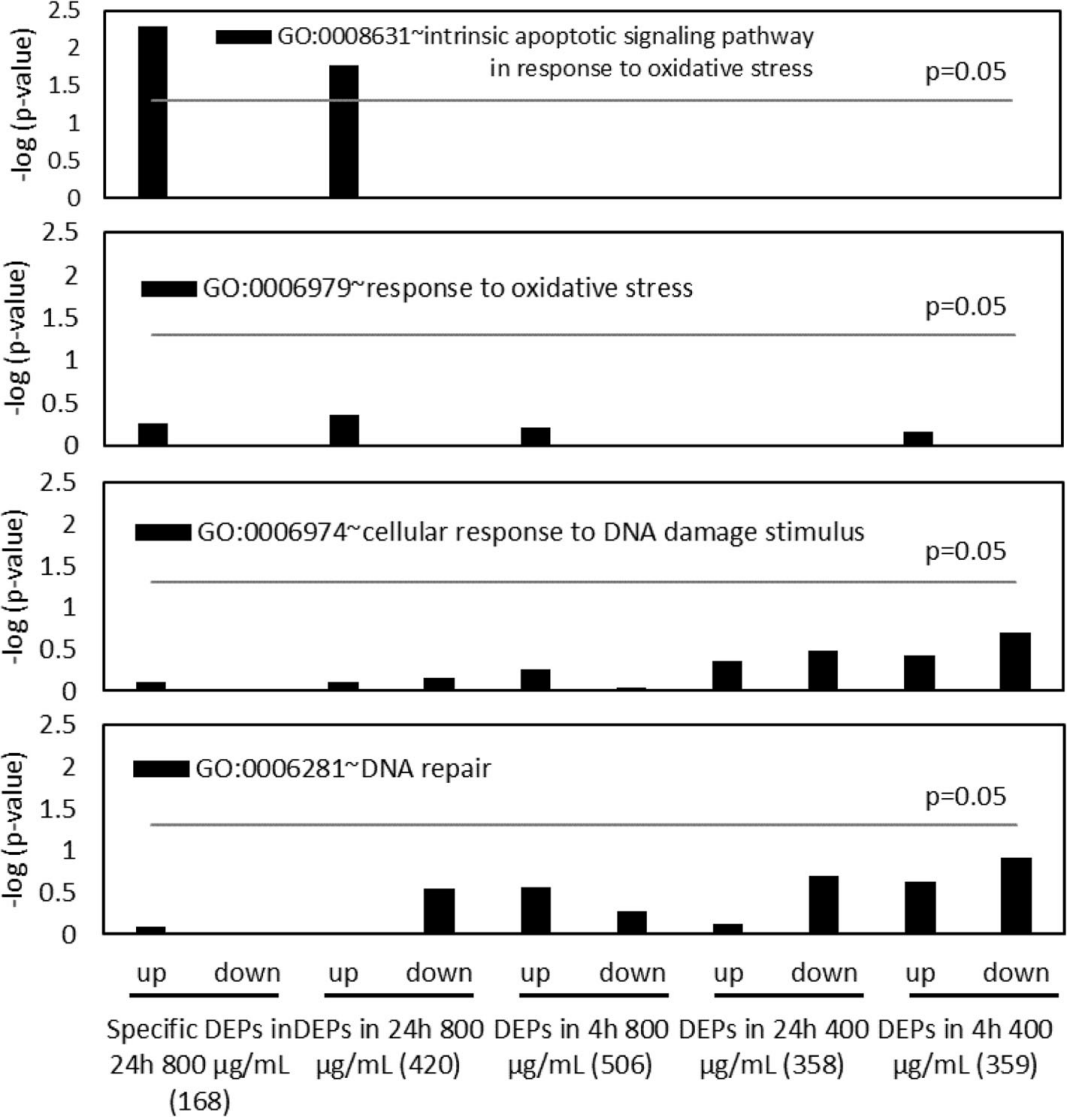
Target gene ontology analysis for TK6 cells exposed to 4-nitroanthranilic acid for 24 h. Whereas no significance was found in GO:0006974 (cellular response to DNA damage stimulus), GO:0006281 (DNA repair), and GO:0006979 (response to oxidative stress), statistically significance was found in GO:0008631 ~ intrinsic apoptotic signaling pathway in response to oxidative stress among DEPs in 24 h 800 μg/mL group and specific DEPs in 24 h 800 μg/mL group, indicating involvement with oxidative stress

In the target analysis of specific GO term among groups (Fig. 3), *P*-values of GO:0006974 (cellular response to DNA damage stimulus), GO:0006979 (response to oxidative stress), and GO:0006281 (DNA repair) were less than 0.05, indicating no significant difference. Conversely, enrichment scores (−log (*p*-value)) of GO:0008631 ~ intrinsic apoptotic signaling pathway in response to oxidative stress were drastically increased in DEPs in 24 h 800 μg/mL group and specific DEPs in 24 h 800 μg/mL group, indicating involvement with oxidative stress.

**Fig. 4:**
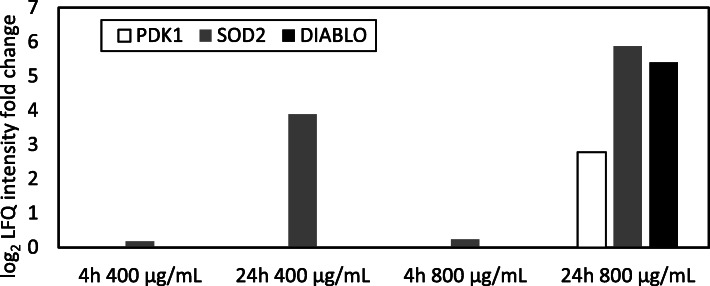
Protein expression levels of the intrinsic apoptotic signaling pathway in response to oxidative stress by the 24 h treatment of 4-nitroanthranilic acid. PDK1, SOD2, DIABLO that belong to GO:0008631 ~ intrinsic apoptotic signaling pathway in response to oxidative stress were drastically elevated only at the 24 h treatment of 4-nitroanthranilic acid, especially in the 800 μg/mL group

In DEPs in 24 h 800 μg/mL group, three proteins ([Pyruvate dehydrogenase (acetyl-transferring)] kinase isozyme 1, mitochondrial phosphoinositide-dependent kinase 1: PDK1, superoxide dismutase 2, mitochondrial: SOD2, direct IAP-binding protein with low PI: DIABLO) that belong to GO:0008631 ~ intrinsic apoptotic signaling pathway in response to oxidative stress were included (Figs. 3 and 4). The expression level of these proteins is elevated only after the 24 h treatment. Furthermore, the expression level of SOD2 was elevated dose-dependently, and PDK1 and DIABLO were only elevated at 800 μg/mL.

## Discussion

### Usefulness of the TK6 assay as a follow-up for testing Ames-positive compounds

The 10 test substances used in this collaborative study were mostly polycyclic aromatic compounds containing amino groups or nitro groups. Characteristics of chemical structures were examined for any regularity or correlation with the results of TK6 assays. The assay can certainly be positive for 8-hydroxyquinoline, 1-nitronaphthalene, and 4-nitro-*o*-phenylenediamine with specific activity values exceeding 10,000 revertants per milligram obtained from Ames test, even three compounds (4-(chloroacetyl)-acetanilide, 2-(chloromethyl) pyridine HCl, and HC Blue No. 2) with small specific activity values of several hundred revertants per milligram were detected as positive (Tables 2 and 3). Thus, the results of TK6 assays are not related to the strength of the specific activity value of the Ames test. Based on our results obtained in the present study, no regularity could be found between mutagenicity results and chemical structures of test substances.

The TK6 assay showed that two substances, 2,5-diaminotoluene and iodoform, were negative (20% effective as a follow-up test), and the remaining eight substances were positive (Table 3). Seven of these substances were reported by MLA using L5178Y cells (Table 3), and all seven showed positive results, including an “inconclusive” judgment for 2,5-diaminotoluene that was reported negative and positive by the Scientific Committee on Consumer Safety (SCCS) [46] and the National Toxicology Program (NTP) database [54], respectively. Compared to p53-proficient TK6 cells, p53-deficient L5178Y cells are more sensitive to spindle inhibitors due to disruption of spindle checkpoints and apoptosis [55]. Moreover, Whitwell et al. reported that the use of human TK6 cells may be preferable over rodent L5178Y cells to help reduce false positive results in in vitro assay [56]. We believe that the TK6 assay, due to negative here for 2,5-diaminotoluene in this study, may indicate a possible improvement over MLA to help follow-up on false-positive results from Ames testing.

In general, it is difficult to follow up the positive results of the Ames test, which detects DNA-reactive substances, with in vitro mammalian cell gene mutation test, as it may lead to similar results to the Ames test in terms of the principle of detecting mutations [4, 57]. In this present study, 20% effective (2 negative results of 10 substances in the TK6 assay) suggests that the TK6 assay may be able to follow up on the positive results of the Ames test. Six substances (4-(chloroacetyl)-acetanilide, 2-(chloromethyl) pyridine HCl, 2,6-diaminotoluene, 8-hydroxyquinoline, 1-nitronaphthalene, and 4-nitro-o-phenylenediamine) that are negative in in vivo genotoxicity tests (chromosomal Aberration test, micronucleus test, and transgenic rodent gene mutation assay) are not considered to be carcinogenic [48, 58–63], because their genotoxicity is eliminated by biological reactions (e.g., ADME; absorption, distribution, metabolism and excretion) even if they show DNA reactivity in vitro such as TK6 assay. Thus, Ames-positive substances, such as 2,5-diaminotoluene, that are negative in the TK6 assay, not via ADME, and negative in the in vivo test may have bacterial-specific DNA reactivity. Kirkland et al. [14, 15] reported that, in the case of an Ames-positive chemical, negative results in two in vitro mammalian cell tests covering both mutation and clastogenicity/aneugenicity endpoints should be considered as indicative of absence of in vivo genotoxic or carcinogenic potential. As the mutation endpoint, the human TK6 assay, even by current methods that have not been combined with new technologies such as proteomics, may have sufficient potential to reduce some false positive results (20%) as a follow-up test.

### Possible mechanisms of chemical mutagenesis involving reactive oxygen species (ROS) production in the long-term treatment with TK6 cells

Interestingly, three (2,6-diaminotoluene, HC Blue No. 2, and 4-nitroanthranilic acid) of the eight positive substances in the present study were negative after the short-term treatment and positive after the 24 h treatment (Table 3), despite identical treatment conditions in the absence of S9. Thus, an extended treatment time of only 20 h long, the judgment of these substances was changed from negative to positive (Table 3). Based on the chemical structures of these three compounds, we discussed the mechanisms of positives results after the long-term treatment in the absence of S9.

#### 2,6-Diaminotoluene

**Fig. 5:**
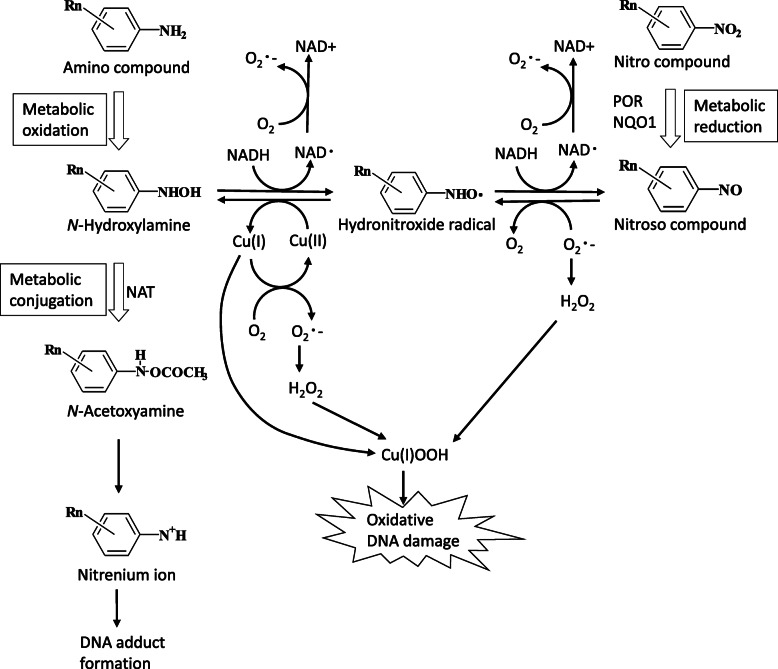
Possible mechanisms of oxidative stress produced by the redox reaction of amino- and nitro compounds in human TK6 cells

Metabolism of hepatic microsomes in general (such as +S9) oxidize arylamines to hydroxylamines by P450 and *O*-acetylized to acetoxyarylamines with acetyltransferase. These further break down spontaneously into arylnitrenium ions that form adducts with bases in nucleic acids [64, 65]. Additionally, arylamines are metabolized to hydroxylamines with amine *N*-oxidase and flavin-containing monooxygenase (FMO) [66]. Mutagenicity was confirmed in human TK6 cells after 96 h of exposure to benzidine, one of the arylamines, in the absence of S9 [67]. Additionally, the mutagenicity of 2,6-diaminotoluene occurs in MLA using L5178Y mouse lymphoma cells (4 h, −S9) [68]. A slight expression of P450 has been reported in TK6 cells [69]. In the present study, the enzyme expression for P450s, amine *N*-oxidase, and FMO was not detected; however, the formation of proteins related to *O*-acetylization (*N*-acetyltransferase) was measured in the proteomic analysis of vehicle TK6 control (Additional file 3 (Table S3)). These findings suggest the possibility of DNA adduct formation by arylnitrenium ions via oxidation of arylamine with some enzymes in TK6 cells (24 h, −S9) [65]. Furthermore, the induction of DNA damage mediated by ROS is highly plausible (Fig. 5) [65, 70–72].

2,4-Diaminotoluene and 2,6-diaminotoluene are considered carcinogenic and non-carcinogenic, respectively, based on a report of the National Toxicology Program (NTP), USA [24], although one study indicated that it is not at all clear that 2,6-diaminotoluene is non-carcinogenic [73]. Significant DNA adducts were detected in the livers of rats exposed to 2,4-diaminotoluene; otherwise, a marginal amount of adducts was detected in the case 2,6-diaminotoluene [74]. In studies on the comparison with 2,4-diaminotoluene, negative results for 2,6-diaminotoluene with respect to hepatocellular proliferation in rats [75], an unscheduled DNA synthesis (UDS) assay in rats [76], a mutagenic assay with LacI transgenic mice [77], and a guanine-hypoxanthine phosphoribosyltransferase (*gpt*) mutagenic assay with transgenic rats [23, 78] have been reported, although positive results were also confirmed at a high dose of 2,6-diaminotoluene via a UDS assay [79]. Furthermore, the negative result was confirmed by a comet assay of various mouse organs [80]. 2,6-Diaminotoluene might be more efficiently detoxified than 2,4-diaminotoluene in vivo because its *para* site at position 4 can be oxidized and subsequently conjugated by phase II enzymes [81]. The formation of the DNA adduct via the nitrenium ion is important for the Ames assay, and it has been reported that the mutagenicity (+S9) of 2,5-diaminotoluene is lower than that of 2,6-diaminotoluene [82]. In the present study, the results showed that 2,5-diaminotoluene was not mutagenic and 2,6-diaminotoluene was mutagenic according to the TK assay in the 4-h treatment with S9 and in the 24-h treatment without S9, which was assumed to affect the formation of the DNA adducts via the arylnitrenium ion.

ROS are formed and mediated by oxidation to quinonediimines in keratinocytes, in which the expression of oxidative enzymes is minimal for arylamines [70]. Additionally, ROS are formed via hydroxylamines and aminophenols, which are P450-mediated oxidative metabolites for arylamines [65, 71, 72]. For arylamines such as 2,6-dimethylaniline and 3,5-dimethylaniline, the principal mechanism of mutagenic action is likely associated with aminophenol/quinone imine redox cycling to generate ROS rather than the formation of covalent DNA adducts via the arylnitrenium ion [71, 72]. 2,6-diaminoltoluene may be metabolized in the same pathway as arylamines. Metabolites of 2,6-diaminoltoluene that contribute to ROS formation and accumulation are unknown.

#### HC Blue No. 2

Nitro compounds are reduced to hydroxylamines with nitroreductases, such as NAD(P) H quinone oxidoreductase (NQO1) and P450 oxidoreductase (POR), and are *O*-acetylized to acetoxyarylamines with acetyltransferase. These metabolites break down spontaneously into arylnitrenium ions that form adducts with bases in nucleic acids [64, 65]. NQO1 is expressed in TK6 in the normal state [83], and the proteomics analysis in the present study, measured nitroreductases, such as NQO1 and POR, and proteins related to *O*-acetylization (*N*-acetyltransferase) in the vehicle TK6 control (Additional file 3 (Table S3)). DNA adduct formation via the arylnitrenium ion and ROS formation via hydroxylamine likely contribute to DNA damage caused by HC Blue No. 2 in TK6 cells after 24 h of exposure in the absence of S9 (Fig. 5) [65].

HC blue No. 1 has been determined to be a carcinogen, whereas HC blue No.2 has been classified as a non-carcinogenic. However, reinterpretation of bioassays showed that the results did not justify HC blue No. 2 being classified as a carcinogenic or as definitely a non-carcinogenic [84]. The MLA (4 h) for HC blue No. 2 was positive in the presence of S9 [27, 29, 68], and the mutagenicity in MLA (+S9) for carcinogenic HC blue No. 1 has also been reported to be positive with weak response [68]. Meanwhile, a negative result has been reported for carcinogenic HC blue No. 1 with high purity in MLA (−S9) and other mutagenic assays [27, 85]. For HC blue No. 2 with high purity, a positive result was obtained in MLA (−S9) though the evaluation of carcinogenicity has not been reported [27]. The differences between in vitro and in vivo metabolites may be responsible for the different results of HC blue No. 1 related to mutagenicity (negative in the absence of S9) and carcinogenicity (carcinogenic) [27]. The in vivo metabolite of HC blue No. 2 was single, and the non-carcinogenic result of HC blue No. 2 in animal experiments may be due to the conjugation and the excretion of the metabolite [27].

#### 4-Nitroanthranilic acid

This chemical possesses three functional groups: nitro, amino, and carboxyl. However, it is presumably metabolized and excreted relatively quickly because of the carboxyl group. Therefore, a negative result of chemical in TK6 assays (4 h, +S9) in the present study was likely due to the facilitation of its oxidative metabolism. Furthermore, 4-nitroanthranilic acid showed a negative result upon exposure for 4 h and a positive result after 24 h in the absence of S9. DNA damage was likely induced by the arylnitrenium ion and ROS via the reductive metabolism of the nitro group that required longer time frames 24 h (Fig. 5). Major oxidative enzymes that act on amino groups, such as P450s, amine N-oxidase, and FMO, were not confirmed, and nitro reductive enzymes, such as NQO1 and POR, were detected via proteomic analysis of TK6 control cells (Additional file 3 (Table S3)) [65, 69, 83].

### Oxidative stress revealed by proteomics analysis

The enrichment analysis of DEPs in the 24 h treatment condition suggested that oxidative stress plays a key role in the enhancement of cytotoxicity and genotoxicity in continuous exposure conditions (Fig. 4). PDK1, SOD2, and DIABLO were expressed prominently after a 24 h exposure of cells to 800 μg/mL 4-nitroanthranilic acid in the present study. These proteins are all involved in response to oxidative stress. A dose- and time-dependent increasing tendency of major antioxidant enzymes (catalase and GSR) was confirmed in the proteomics analysis (Additional file 3 (Table S3) and 4 (Figure S1)). After the short-term treatment, antioxidant defenses such as glutathione and related enzymes in TK6 cells were able to suppress ROS damage, but with the long-term treatment, the increase of oxidative stress caused by depletion of antioxidant enzymes is expected. The prominent decrease of several antioxidant enzymes (GPX4, MGST3, TXN2, TXNRD1, etc.) at 24 h in contrast to 4 h in the proteomics analysis may reflect such depletion (Additional file 3 (Table S3) and 4 (Figure S1)). Additionally, oxidative stress has been considered as a common underlying mechanism for in vitro specific genotoxicity. Generally, mammalian tissues in vivo are likely to display greater antioxidant defenses than cells in culture. Chemicals that induce genotoxicity via the production of ROS, in such case, would damage DNA directly but would be expected to have a threshold [86]. Further analyses are needed to confirm whether these same features of DEPs are observed in other chemicals with conflicting test results. If the same features are observed, quantification of these proteins (or genes) or ROS would provide a promising solution to discriminating in vitro specific positive results from a mode of action point of view.

## Conclusions

The usefulness of the TK6 assay, by current methods that have not been combined with new technologies such as proteomics, was found to be limited as a follow-up test, although it still may help to reduce some false positive results (20%) in Ames tests. Furthermore, in vitro specific genotoxicity was prominently exhibited in the long-term treatment, but proteomics analysis of a false-positive compound revealed a mode of action, in which genotoxicity caused was induced by oxidative stress during the long-term treatment. Therefore, an integration of omics technology and TK6 assays may contribute to interpreting irregular results in 24 h specific positives and might lead to further reduction (> 20%) of false positives in follow-up to Ames tests.

## Supplementary Information

**Additional file 1.**

**Additional file 2.**

**Additional file 3.**

**Additional file 4.**

## Data Availability

Not applicable.

## Abbreviations

TK6 assay: Thymidine kinase gene mutation assay
WoE: Weight of evidence
JEMS: The Japanese Environmental Mutagen Society
MMS: The Mammalian Mutagenicity Study group
OECD: The Organization for Economic Co-operation and Development
AOP: Adverse outcome pathway
IATA: Integrated approaches to testing and assessment
SCCS: Scientific Committee on Consumer Safety
MLA: Mouse lymphoma assay
CE: Cloning efficiency
RS: Relative survival
RSG: Relative suspension growth
RTG: Relative total growth
MF: Mutant frequency
LFQ: Label-free quantification
TCEP: Tris (2-carboxyethyl)phosphine
NaN: Not a number
DEP: Differentially expressed protein
GO: Gene Ontology
