# Supplementary figures and images for: Weight of evidence approach using a TK gene mutation assay with human TK6 cells for follow-up of positive results in Ames tests: a collaborative study by MMS/JEMS
Source: Genes Environ. 2021 Mar 6;43:7. doi: 10.1186/s41021-021-00179-1 (PMC7937321; doi:10.1186/s41021-021-00179-1)

## Slide 1
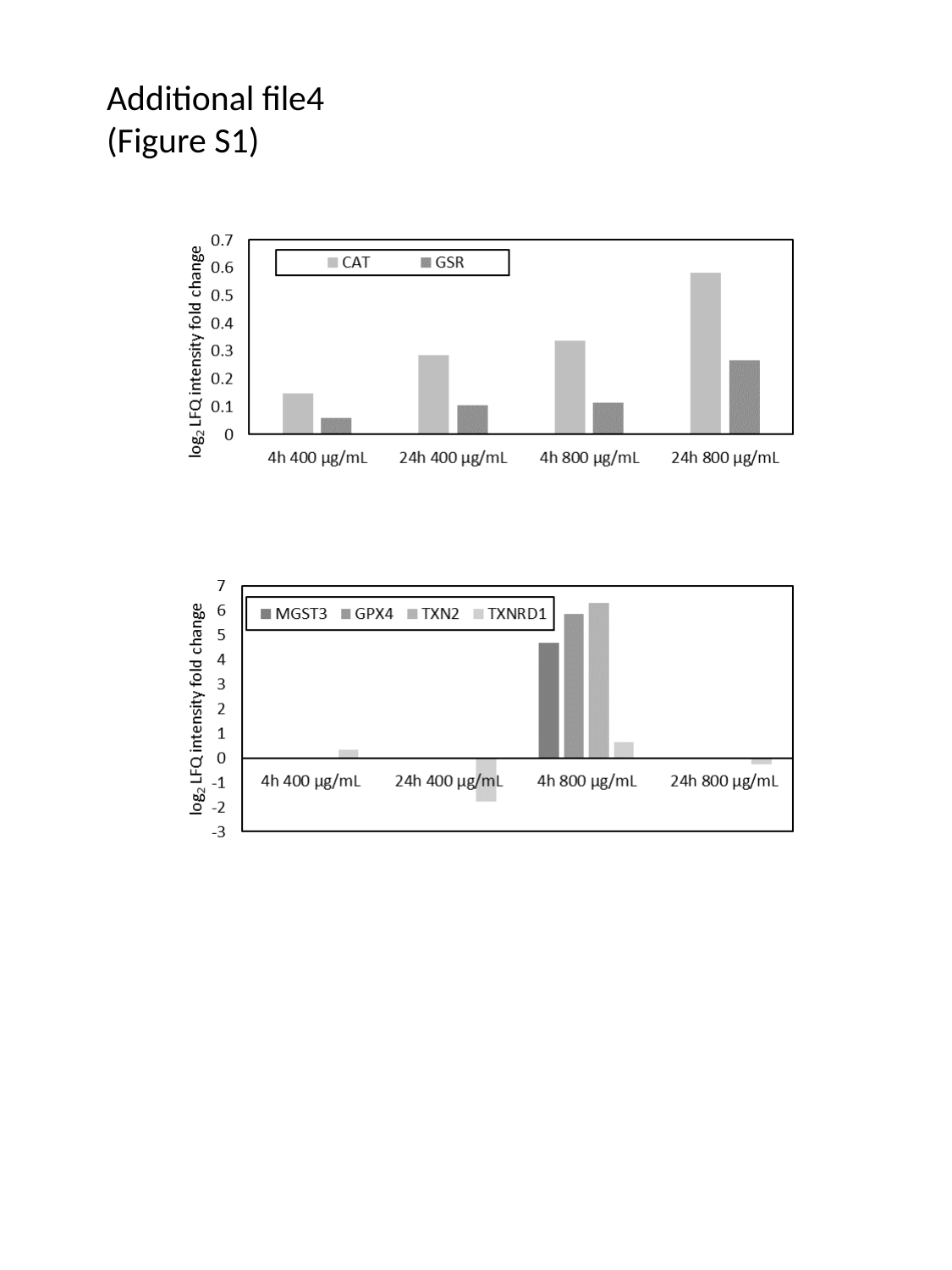

Additional file4 (Figure S1)

Supplement: Supplementary file 4 — Additional file 4. [file 41021_2021_179_MOESM4_ESM.pptx]
